# Low-Temperature Stress-Induced Hepatic Injury in Darkbarbel Catfish (*Pelteobagrus vachelli*): Mediated by Gut–Liver Axis Dysregulation

**DOI:** 10.3390/antiox14070762

**Published:** 2025-06-21

**Authors:** Amei Liu, Guoqing Duan, Libo Yang, Yuting Hu, Huaxing Zhou, Huan Wang

**Affiliations:** 1 Institute of Fisheries Science, Anhui Academy of Agricultural Sciences, Nongke South Road, Hefei 230031, China; liuamei@aaas.org.cn (A.L.); huyuting@aaas.org.cn (Y.H.); zhouhuaxing@aaas.org.cn (H.Z.); wanghuan@aaas.org.cn (H.W.); 2 Anhui Province Key Laboratory of Aquaculture & Stock Enhancement, Hefei 230031, China; 3 Institute of Artificial Intelligence, Hefei Comprehensive National Science Center, Hefei 230088, China; yanglibo1212@iai.ustc.edu.cn

**Keywords:** low-temperature stress, darkbarbel catfish (*Pelteobagrus vachelli*), antioxidants, gut microbiota, metabolome, gut–liver axis

## Abstract

Low-temperature stress serves as a critical abiotic stressor that severely restricts fish survival, biogeographic distribution, and aquaculture productivity. *Pelteobagrus vachelli*, an economically significant freshwater fish species, displays marked sensitivity to low-temperature stress; however, its molecular adaptive mechanisms remain poorly characterized. In this study, we systematically investigated hepatic and intestinal cold stress responses in *P. vachelli* through a 7-day acute low-temperature exposure trial (6 °C and 11 °C), integrating histopathological examination, physiological–biochemical assays, metabolomics, and 16S rRNA sequencing. Histopathological observations revealed pronounced hepatic vacuolar degeneration, nuclear dissolution, and enhanced inflammatory cell infiltration under low-temperature conditions. Concurrently, immune-related enzymatic activities—including aspartate aminotransferase (AST), alanine aminotransferase (ALT), and alkaline phosphatase (APK)—were significantly elevated. Furthermore, substantial perturbations in antioxidant defense systems were detected, as indicated by altered superoxide dismutase (SOD) and glutathione peroxidase (GSH-Px) activities, alongside malondialdehyde (MDA) accumulation. Metabolomic profiling identified 539 differentially abundant metabolites, with pathway enrichment analysis highlighting marked alterations in FoxO signaling, amino acid metabolism, glycerophospholipid metabolism, ABC transporter, and Purine metabolism. Gut microbiome sequencing demonstrated cold-induced structural dysbiosis within the intestinal microbiota. Correlation analyses revealed robust linkages between hepatic injury biomarkers/metabolites and specific intestinal microbial taxa. Collectively, this study delineates the interplay between hepatic metabolic reprogramming and gut microbiota dysbiosis during cold adaptation in *P. vachelli*, establishing a theoretical framework for developing gut–liver axis-targeted strategies to augment cold tolerance in aquatic species.

## 1. Introduction

In recent years, global climate change has intensified seasonal temperature fluctuations and extreme events, threatening aquatic ecosystems [1,2,3]. As poikilotherms, fish physiology and metabolism are tightly linked to water temperature [4,5], making their responses to thermal stress a critical research focus. Consequently, elucidating the physiological response mechanisms underlying teleost adaptation to extreme thermal stressors has emerged as a critical research priority in aquatic sciences. Studies have demonstrated that acute cold stress elicits a cascade of physiological and pathological responses in fish, including feeding suppression [6,7], gut microbiota dysbiosis [8,9], energy metabolic perturbation [10,11], and imbalances in tissue antioxidant–immune systems [9,12]. These low-temperature stress-induced alterations severely threaten both the ecological stability of cultured populations and their economic value in aquaculture industries.

The liver, as the primary metabolic organ in vertebrate, plays a pivotal role in regulating energy homeostasis, immune defense, and detoxification metabolism in fish. Recent studies have revealed that cold stress induces histopathological alterations and metabolic dysregulation in fish hepatic tissues [4,13,14]. Specifically, hepatocyte vacuolization and glycogen depletion were observed in black porgy (*Acanthopagrus schlegelii*) under low-temperature conditions [4], while silver pomfret (*Pampus argenteus*) exhibited more pronounced hepatic lipid accumulation, cytolysis, and concomitant disturbances in amino acid and fatty acid metabolism [15]. Moreover, the intestinal system—serving as the primary interface for nutrient absorption and immunological defense—demonstrates particular sensitivity to thermal fluctuations in terms of its morphological integrity [16], microbial community homeostasis [17], and immunomodulatory capacity [18]. Experimental evidence reveals that low-temperature exposure induces increased intestinal permeability in large yellow croaker (*Larimichthys crocea*) [19], while freshwater drum (*Aplodinotus grunniens*) [20] exhibits ultrastructural alterations, including microvilli atrophy, mitochondrial deformation, and tight junction disruption. Yellow catfish (*Pelteobagrus fulvidraco*) further display marked reductions in gut microbiota α-diversity with concurrent proliferation of opportunistic pathogens [21].

Although many studies have confirmed the independent damaging effects of low temperatures on the hepatointestinal system in aquatic organisms, the inter-organ communication mechanisms remain poorly characterized. The gut–liver axis delineates a bidirectional regulatory framework wherein microbial metabolites modulate hepatic functions via the portal vein [22], while hepatobiliary-derived bile acids reciprocally reshape gut microbial composition a cross-talk mechanism extensively documented in mammalian energy metabolism and immune regulation [23,24]. Central to this regulatory network is the process of hepatic remodeling—a dynamic adaptation involving both structural and functional reorganization of liver tissue. In aquatic species, microbial dysbiosis can trigger characteristic remodeling features, including inflammation, fibrosis, and metabolic reprogramming, as demonstrated in zebrafish [25]. Similar observations in grass carp [26] have established direct links between gut-derived metabolites (e.g., short-chain fatty acids) and hepatocyte proliferation/lipid metabolism. Furthermore, oxidative stress-induced hepatic remodeling has been documented in swamp eel [27], highlighting the evolutionary conservation of microbiome-liver crosstalk in aquaculture species. Notwithstanding these advances, the regulatory networks governing gut–liver interactions, microbial–host metabolic crosstalk, and tissue repair mechanisms in ectothermic animals (particularly fish) under temperature stress remain largely unexplored. These knowledge gaps justify our focused investigation on hepatic remodeling as a key integrative process in the gut–microbiome–liver axis.

*Pelteobagrus vachelli*, a commercially important freshwater fish species in China, holds significant aquacultural value due to its high economic potential [28,29]. Previous studies on *P. vachelli* have primarily focused on aquacultural aspects, including dietary lipid optimization for growth [30] and immune responses to bacterial infection [31]. As a thermophilic species, its optimal growth temperature ranges from 20 to 32 °C [32]. However, when environmental temperatures drop below 13 °C, this species exhibits pronounced cold stress responses, including behavioral suppression [21]. Notably, during winter cold snaps in northern China, such temperature declines frequently induce mass mortality events in aquaculture populations, resulting in substantial economic losses to the industry. Therefore, an in-depth exploration of the low-temperature response mechanisms in *P. vachelli* holds significant theoretical and practical importance.

This study employed *P. vachelli* as the experimental model and established acute low-temperature stress models at 6 °C and 11 °C. A multi-omics integrative analysis strategy was systematically implemented to conduct the following investigations: (1) utilizing histopathology and enzyme-linked immunosorbent assay (ELISA), the dynamic changes in hepatic tissue structural damage and antioxidant/immune functions were elucidated; (2) untargeted metabolomics was applied to delineate the characteristics of hepatic metabolic remodeling and identify key metabolic pathways under low-temperature stress; (3) 16S rRNA high-throughput sequencing was employed to reveal the response patterns of intestinal microbial community structure; (4) metabolomic and microbiomic datasets were integrated to construct an intestine–liver axis interaction network model. Innovatively, this research deciphers the molecular mechanisms of low-temperature stress in fish from the perspective of gut–liver axis regulation, not only providing novel scientific insights for refining environmental adaptation theories in poikilothermic animals but also establishing a theoretical foundation for developing winter health management strategies in aquaculture species.

## 2. Materials and Methods

### 2.1. Fish and Experimental Design

**Figure 1:**
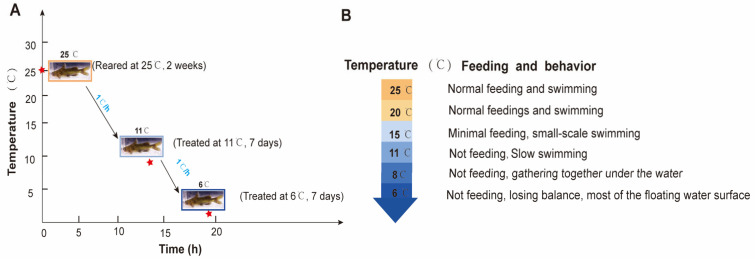
The experimental design and behavior performance for *P. vachelli* under hypothermic stress. (**A**) Schematic diagram of the experimental design for *P. vachelli* sampling under cold stress conditions. The red asterisks denote the time points of temperature treatments: the temperature was decreased at a rate of 1 °C per hour (from 25 °C to 11 °C: 14 h; from 11 °C to 6 °C: 5 h). (**B**) Statistical analysis of behavioral responses in *P. vachelli* at different temperature points.

A total of 270 *P. vachelli* (average body weight: 46.32 ± 5.67 g; length: 17.56 ± 0.78 cm) were collected from the Fisheries Research Institute of Anhui Academy of Agricultural Sciences. The fish were randomly distributed into nine tanks (30 fish per tank) in a recirculating water system (temperature: 25 ± 1 °C; dissolved oxygen: 7.0 ± 0.5 mg/L; pH: 7.3 ± 0.5) and acclimated for two weeks. During acclimation, the fish were fed commercial feed at 2% (*w*/*w*) of their body weight twice daily. In the experiment, 25 °C (C) served as the control temperature, while 11 ± 1 °C (T1) and 6 ± 1.5 °C (T2) were used as low-temperature treatments for seven days. The temperature was gradually decreased from 25 °C to 11 °C and 6 °C at a rate of 1 °C/h. Feeding behavior was evaluated by quantifying uneaten pellets as a percentage of the total feed provided. Fish were acclimated to each target temperature (from 25 °C to 6 °C, with a gradual decrease of 1 °C per hour) in triplicate tanks (one tank per temperature group for preliminary observation). Pellets were offered twice daily at fixed times, and uneaten portions were collected after 30 min to calculate feeding rates. Behavioral responses (activity levels, aggregation patterns) were recorded daily via direct observation (Figure 1A). The water temperature was stabilized at the set low temperatures. During the experiment, uneaten feed and feces were removed daily.

### 2.2. Sample Collection

Experimental samples were collected seven days after treatment. Nine *P. vachelli* from each group (3 tanks × 3 fish = 9 fish total per group) were randomly selected and anesthetized with 0.02% tricaine methanesulfonate (MS-222). Liver samples were collected from 9 selected fish in each group for biochemical analyses and metabolome analysis. Intestinal content samples were harvested from the same fish for metagenomic analysis of the gut microbiota (Figure S1). All samples were immediately frozen in liquid nitrogen and then stored at −80 °C for further experiments. Subsequently, liver samples from 3 fish were rapidly placed in 4% paraformaldehyde solution for histological analyses.

### 2.3. Histological and Structural Observations

Hematoxylin–eosin (H&E) staining was performed on the liver tissue samples. The samples were fixed in 4% paraformaldehyde for 48 h, and then dehydrated in a stepwise ethanol gradient concentration of ethanol (*v*/*v*: 50% ethanol, 75% ethanol, 95% ethanol, and anhydrous ethanol) and permeabilized in xylene, embedded in paraffin, and then the tissues were cut into 5 μm sections and stained with H&E [33]. The stained sections were observed and photographed under an optical microscope (Olympus, Tokyo, Japan). Nucleus area and vacuolation were quantified using ImageJ software (NIH imageJ, Version 2, Bethesda, MD, USA) by measuring pixel intensity and vacuole-to-cytoplasm ratio in 10 randomly selected cells per tissue section (n = 3 sections per sample). A total of 30 cells per treatment group were analyzed.

### 2.4. Determination of Antioxidant Enzyme Activities

The liver tissue samples were mechanically homogenized in a pre-cooled physiological saline solution (0.9% NaCl) at a ratio of 1:9 (*w*/*v*) in ice water-bath conditions to prepare for 10% homogenization solution, respectively. The obtained homogenate was then centrifuged for 10 min on 3500× *g* at 4 °C, and then the supernatant was collected for biochemical analysis. The enzyme activities of superoxide dismutase (SOD), reduced glutathione (GSH), Malondialdehyde (MDA), alkaline phosphatase (AKP), and Alanine Aminotransferase (ALT). SOD, GSH, and MDA, as well as other enzyme activities were determined using commercial kits (Beyotime Biotechnology, Shanghai, China). Three biological replicates were set for each treatment group, and the operation method was performed according to the operating instructions. Before measuring the above parameters, the total protein of the sample concentration was quantified using the Coomassie Brilliant Blue method, performed according to the kit [13,20].

### 2.5. Gut Microbiome Analysis

The microbial genomic DNA of *P. vachelli* was extracted with using the DNA Kit (Omega Bio-tek, Norcross, GA, USA). Then, the V3–V4 region of the 16S rRNA gene was selected as a biomarker, and the PCR reagents and cycling parameters were the same as previously described [29]. After, the amplicons were quantified, following which the normalized equimolar concentrations of amplicons were pooled and sequenced on and sequenced on the Illumina NovaSeq 6000 platform (Illumina Inc., San Diego, CA, USA) with PE150 sequencing mode according to the manufacturer’s specifications. The microbial diversity and differences in microbial community compositions of samples were analyzed via taxonomic composition and the alpha/beta diversity analysis. The differences in the relative abundances at the genus levels between the low-temperature stress group and control group were tested using one-way ANOVA, and a difference of *p* < 0.05 was considered significant. The functional profile of gut microbiota was predicted using a PICRUSt2 analysis based on the KEGG metabolic pathway analysis. The Linear Discriminant Analysis Effect Size (LEfSe) analysis was conducted via the Galaxy web platform (https://huttenhower.sph.harvard.edu/galaxy/, accessed on 19 March 2025), with an LDA (Linear Discriminant Analysis) score threshold set to 4.0.

### 2.6. Metabolomic Analysis

Three biological replicates of liver sample from three groups were used for non-targeted LC-MS metabolomic analysis. The metabolites were extracted from the liver for metabolome analysis using UPLC (1290 Infinity LC, Agilent Technologies, Santa Clara, CA, USA) and MS (QTOF/MS-6545, Agilent Technologies, Santa Clara, CA, USA). The three-dimensional dataset comprising peak indices, sample identifiers, and normalized peak areas was analyzed using SIMCA software (version 14.1; Umetrics, MKS Data Analytics Solutions, Umeå, Sweden). Multivariate analysis was conducted through orthogonal partial least squares discriminant analysis (OPLS-DA), complemented by loadings plot interpretation and volcano plot visualization to eliminate inter-group outliers and detect potential biomarkers demonstrating significant inter-group variations. Differential metabolite screening was implemented with dual criteria: variable importance in projection (VIP) scores ≥ 1.0 and adjusted *p*-values < 0.05 (Student’s *t*-test with FDR correction). Metabolic pathway analysis was performed using the Kyoto Encyclopedia of Genes and Genomes (KEGG) database (website: http://www.metaboanalyst.ca, accessed on 15 October 2024). Subsequent pathway enrichment analysis and graphical representations were generated through MetaboAnalyst 5.0 web platform.

### 2.7. Correlation Analysis

Spearman’s rank correlation analysis was performed to assess the associations between differentially abundant gut microbiota genera and hepatic antioxidant/damage parameters. Statistical significance was determined by Benjamini–Hochberg false discovery rate (FDR) correction with q < 0.05. Hierarchical clustering heatmaps were visualized using the pheatmap package (v1.0.12) in the R statistical environment.

### 2.8. Statistical Analyses

All statistical analyses, conducted using GraphPad Prism for Windows (GraphPad Software, Version 7.0, San Diego, CA, USA), were used to analyze the differences among groups. Significant differences between different groups were detected using a one-way analysis of variance (ANOVA) and a Student’s *t*-test, and a *p*-value less than 0.05 was considered significant; the numerical value is expressed as mean ± SEM.

## 3. Results

### 3.1. Effects of Low-Temperature Stress on Feeding Rate and Behavior in Pelteobagrus vachelli

During low-temperature stress, the feeding and swimming abilities of *P. vachelli* exhibited significant alterations (Figure 1). In the initial phase, the fish maintained normal feeding behavior and swimming activity. As the temperature progressively decreased, the adverse effects intensified, leading to a gradual decline in feeding capacity until complete cessation of intake. Concomitant changes in swimming performance were observed, transitioning from normal swimming to slow swimming, then to sideways swimming, and finally lying motionless until overturning occurred (Figure 1B). Reaction ability declined progressively, transitioning from sensitive responses to sluggish behavior. Quantitative analysis revealed that feeding capacity showed a temperature-dependent decrease as temperatures descended from 25 °C to 11 °C, with complete feeding termination occurring below 11 °C. Subsequent cooling from 11 °C to 8 °C induced progressive hypokinesis, culminating in behavioral immobilization and aggregation at the bottom of the tank. Notably, when temperatures reached 6 °C, some specimens displayed loss of equilibrium manifesting as ventral inversion symptoms, indicative of severe negative impact of cold stress.

### 3.2. Effects of Low-Temperature Stress on Liver Histology in Pelteobagrus vachelli

**Figure 2:**
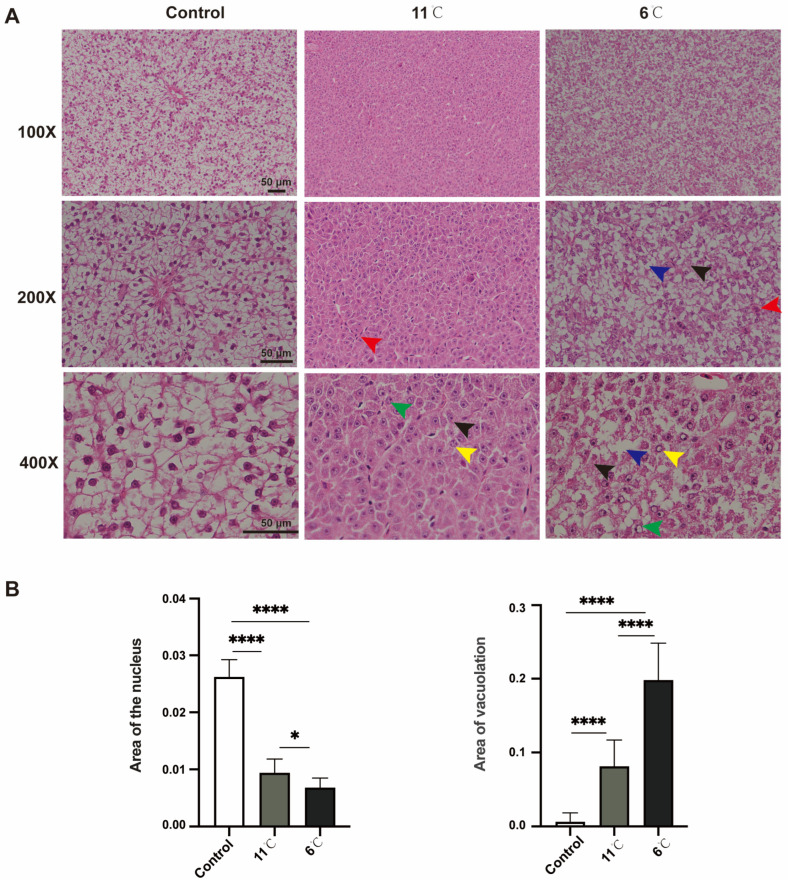
Effects of low-temperature stress on hepatic tissue in *P. vachelli*. (**A**) Histological observations of liver of *P. vachelli* at low-temperature stress and control group with hematoxylin and eosin (H&E) staining. (×100, ×200 and ×400, respectively). Red arrow: focal areas exhibited inflammatory cell infiltration. Blue arrow: hepatocytes demonstrated characteristic ballooning degeneration with cytoplasmic vacuolization. Black arrow: ill-defined cell borders and loosely arranged cytoplasm displaying a reticular pattern. Green arrow: nuclear alterations were observed as pyknotic condensation. Yellow arrow: karyolysis with nuclear disappearance, indicative of programmed cell death processes. (**B**) Area of liver vacuolation and nuclear. Data are mean ± SEM. (n = 10). The * (*p* < 0.05) and **** (*p* < 0.0001) indicate the significant difference between groups.

Histopathological alterations in the liver of *P. vachelli* following 7 days of exposure to different low-temperature stress conditions were observed (Figure 2). H&E staining revealed that the liver tissue of the control group *P. vachelli* had a clear structure. The hepatocytes were ovoid and regularly arranged, with the nuclei located in the center, and the intercellular junctions were clearly visible, with abundant and close arrangements.

In contrast, the 11 °C-exposed group exhibited pathological changes, including the loss of hepatocellular demarcation, peripheral nuclear pyknosis, and perivascular inflammatory cell infiltration. Progressive deterioration was observed under lower temperatures: specimens from the 6 °C-exposed group demonstrated vascular congestion, cytoplasmic vacuolization, ballooning degeneration, and nuclear lysis with complete chromatin dissolution (Figure 2A).

Collectively, these morphological findings demonstrate that sustained cold exposure induces temperature-dependent hepatic damage in *P. vachelli*, with histopathological severity showing direct correlation with thermal reduction magnitude (Figure 2B).

### 3.3. Effects of Low-Temperature Stress on Antioxidant Enzymes and Innate Immunity Biochemical Parameters in Pelteobagrus vachelli

**Figure 3:**
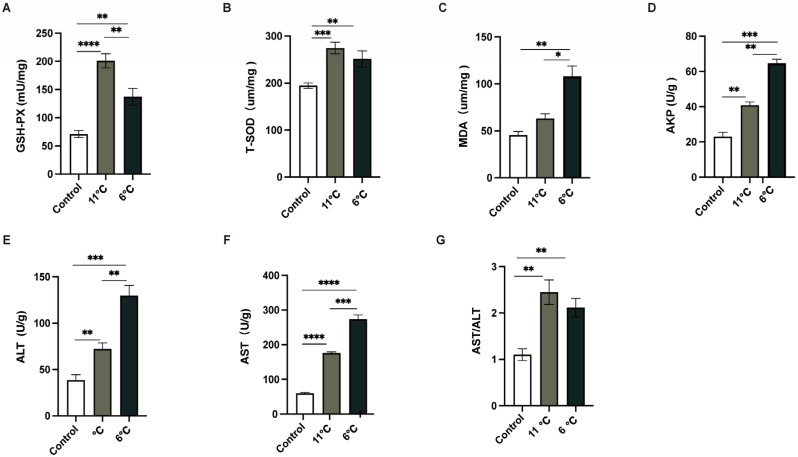
Effects of low-temperature stress on antioxidant and innate immunity. (**A**) Glutathione peroxidase, GSH-Px. (**B**) Total superoxide dismutase, T-SOD. (**C**) Malondialdehyde, MDA. (**D**) Alkaline phosphatase, AKP. (**E**) Alanine aminotransferase, ALT. (**F**) Aspartate aminotransferase, AST. (**G**) The ratio of AST to ALT, AST/ALT. The data were calculated by Students’ *t*-test with GraphPad Prism 9. The * (*p* < 0.05), ** (*p* < 0.01) *** (*p* < 0.001) and **** (*p* < 0.0001) indicate the significant difference between groups. Results are expressed as mean ± SEM.

To comprehensively analyze the liver injury induced by low-temperature stress, the activities of antioxidant enzymes and innate immunity enzymes in the liver were meticulously measured. Three parallel samples were collected from the control group (25 °C) and the low-temperature groups subjected to temperatures of 11 °C and 6 °C. As depicted in (Figure 3), the activities of GSH-PX, T-SOD, and MDA, all of which were closely associated with antioxidant enzyme systems (Figure 3A–C), in *P. vachelli* under low-temperature stress conditions were significantly elevated compared to those in the control group. These findings suggest that an intensified oxidative stress response in the liver under low-temperature exposure. Furthermore, to evaluate the liver injury caused by low temperature, innate immunity enzyme indicators were employed. In contrast to the control group, the activities of AST, ALT, and APK in the liver were notably increased upon exposure to low temperatures (Figure 3D–F).

These alterations in enzyme activities are indicative of potential disruptions in the normal physiological functions of the liver. Collectively, the results of this study provide robust evidence that exposure to low temperatures induces liver damage in *P. vachelli*, as manifested by the significant changes in both antioxidant and innate immunity enzyme activities.

### 3.4. Effects of Low-Temperature Stress on Liver Metabolome in Pelteobagrus vachelli

**Figure 4:**
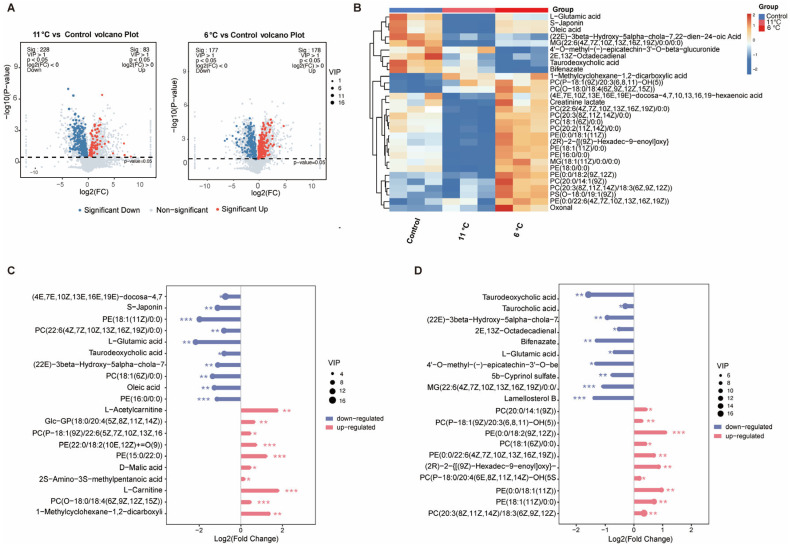
Global analysis of differentially expressed metabolites (DEMs) from the metabolomes of the low-temperature stress and control groups. (**A**) Volcano plot of DEMs in 11 °C/6 °C vs. control comparison. Red and blue represent significantly upregulated and downregulated DEMs, respectively. (**B**) Heatmap of top 30 metabolite clustering analysis. (**C**,**D**) Contrastive visualization of up-/downregulated top 20 differentially expressed metabolites. (**C**) Low-temperature (11 °C) group compared with control group, (**D**), low-temperature (6 °C) group compared with control group. The * (*p* < 0.05), ** (*p* < 0.01) and *** (*p* < 0.001) indicate the significant difference between groups. Results are expressed as mean ± SEM.

To investigate the effects of low-temperature stress on hepatic metabolomics in *P. vachelli*, LC-MS-based metabolomic profiling was performed (Figure 4). Partial least squares discriminant analysis (PLS-DA) revealed significant spatial separation trends among the three comparison groups in two-dimensional space (Figure S2A), indicating that low-temperature stress markedly altered hepatic metabolic patterns. Cross-comparative analysis identified 539 differentially expressed metabolites (DAMs), including 146 common metabolites shared across all three groups (Figure S2B). Volcano plots visually illustrated the quantities of significantly upregulated and downregulated metabolites in each comparison group (Figure 4A). Heatmap analysis further demonstrated distinct metabolic profiles among groups, with the top 30 differentially abundant metabolites primarily encompassing amino acids, flavonoids, fatty acids, bile acids, and phospholipids (Figure 4B). Subsequent analysis of the top 10 most significantly altered metabolites revealed that bile acids, fatty acids, and L-glutamic acid were significantly downregulated in the low-temperature stress group, whereas phospholipids exhibited pronounced upregulation (Figure 4C,D). These compensatory adjustments suggest temperature-adaptive remodeling of hepatic metabolic networks in *P. vachelli*, particularly involving membrane fluidity regulation through phospholipid biosynthesis and energy homeostasis via lipid catabolism suppression.

### 3.5. Analysis of Metabolome Function in Pelteobagrus vachelli Under Low-Temperature Stress

**Figure 5:**
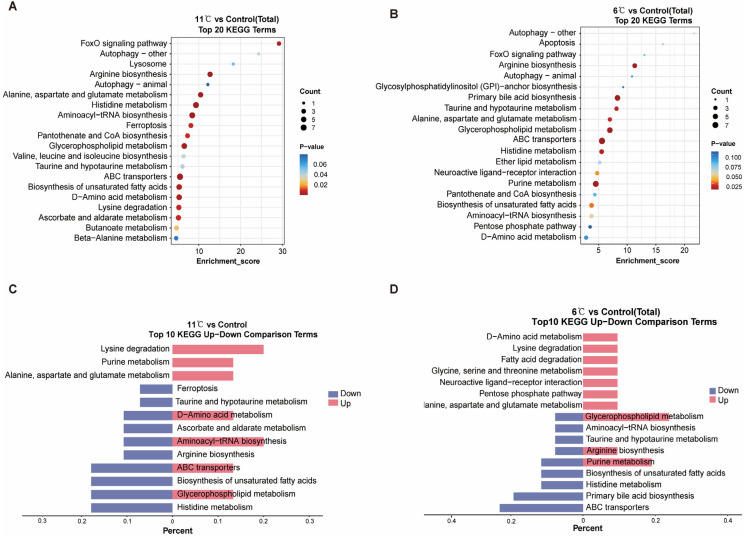
KEGG enrichment analyses of the DEGs in the cold stress and control comparison. (**A**,**B**) KEGG enrichment top 20 pathways of *P. vachelli* under low-temperature temperature stress. (**C**,**D**) Contrastive visualization of up-/downregulated metabolic pathways from KEGG enrichment top10 pathway. (**C**), 11 °C vs. control, (**D**), 6 °C vs. control.

To investigate the functional implications of metabolites exhibiting significant changes in response to low-temperature stress in the liver of *P. vachelli*, Kyoto Encyclopedia of Genes and Genomes (KEGG) enrichment analyses were performed on all DEMs (differentially express metabolites), Enrichment analysis of the top 20 significantly different metabolic pathways revealed notable responses across four major KEGG level 1 categories: “Metabolism”, “Environmental Information Processing”, “Cellular Processes”, and “Genetic Information Processing” (Figure S3A,B). At the KEGG level 2 classification, carbohydrate metabolism, lipid metabolism, and amino acid metabolism pathways were significantly enriched under low-temperature stress within the “Metabolism” category (Figure 5A,B). The “Environmental Information Processing” system was primarily associated with signal transduction pathways, while “Cellular Processes” predominantly involved transport and catabolism pathways. Notably, the “Genetic Information Processing” category showed significant impacts on translation-related pathways, particularly ribosome biogenesis and associated biosynthesis mechanisms.

At KEGG level 3, notably, 11 °C and 6 °C induced distinct stress responses: 11 °C activated FoxO signaling, glycerophospholipid metabolism, and ABC transporters (adaptive energy conservation), while 6 °C uniquely triggered apoptosis, purine metabolism, and GPI-anchor biosynthesis (indicative of severe cellular damage). Shared pathways (e.g., amino acid metabolism) showed divergent regulation: upregulation at 11 °C supported metabolic remodeling, whereas downregulation at 6 °C reflected energy depletion. Interestingly, ABC transporters, purine metabolism, glycerophospholipid metabolism, and amino acid metabolism exhibited significant regulation under both stress conditions, yet manifested divergent expression patterns (Figure 5C,D). Collectively, these findings strongly suggest that different intensities of low-temperature stress prompt organisms to initiate metabolic adaptation strategies. This occurs through gradient-dependent modulation of membrane transport efficiency, facilitated by ABC transporters, and the maintenance of energy homeostasis, achieved via coordinated metabolic reprogramming of the glycerophospholipid, amino acid, and purine metabolism networks.

### 3.6. Effects of Low-Temperature Stress on Gut Microbiome in Pelteobagrus vachelli

**Figure 6:**
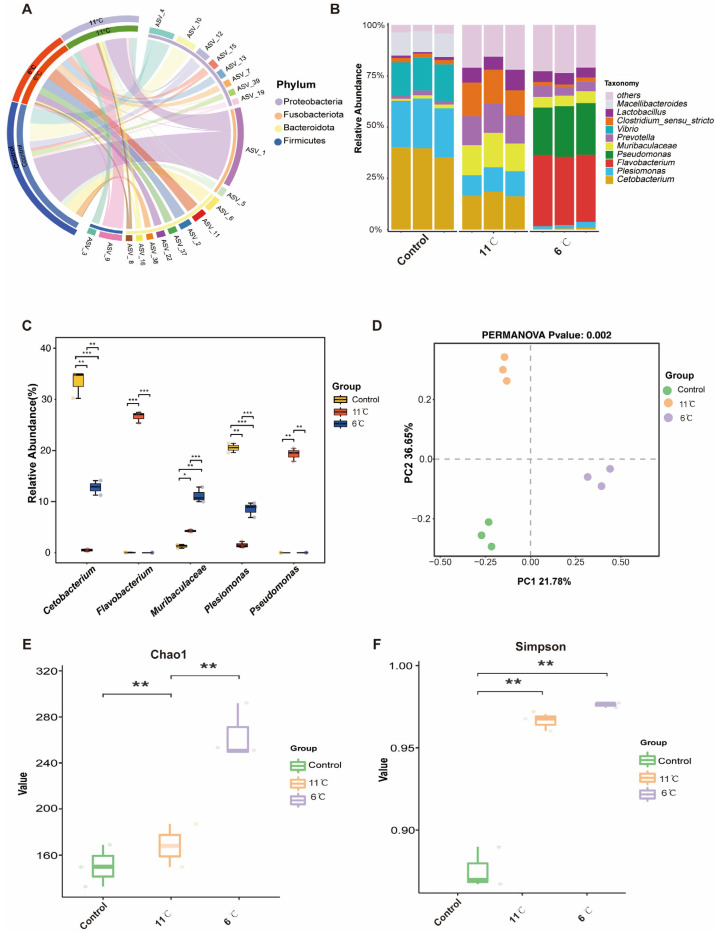
Composition structure and diversity analysis of the gut microbiota. (**A**) Microbial composition at the phylum level. (**B**) Microbial composition at the genus level. (**C**) Statistical analysis of indicated species at the genus level. (**D**) PCA analysis of microorganisms. (**E**) Chao1 index. (**F**) Simpson index. The asterisk represents a significant difference between the two groups. * *p* < 0.05, ** *p* < 0.01, *** *p* < 0.01.

Figure 6A shows an overview of the composition of the microbiome at the phylum level. At the phylum level, Proteobacteria, Firmicutes, and Bacteroidetes were dominant in the gut bacteria of *P. vachelli* (Figure 6A). For genus level, the top five genera, *Cetobacterium*, *Plesiomonas*, *Flavobacterium*, *Pseudomonas*, and *Muribaculaceae*, were found in the intestinal flora of *P. vachelli* (Figure 6B). It was also discovered that the abundances of the genus *Cetobacterium* and *Plesiomonas* flora in the low-temperature stress groups were significantly downregulated compared with the control group. Conversely, the abundances of *Flavobacterium*, *Pseudomonas*, and *Muribaculaceae* flora were significantly increased in the low-temperature stress groups compared with the control group (Figure 6C). Significant differences in beta diversity among the three groups were observed from Principal Component Analysis (PCA) (Figure 6D). The alpha diversity index of the gut microbiota was assessed by the Kruskal–Wallis test (Figure 6E,F). Compared with the control group, the Chao1 and Simpson indexes in the intestinal microflora of *P. vachelli* were significantly increased under low-temperature stress.

**Figure 7:**
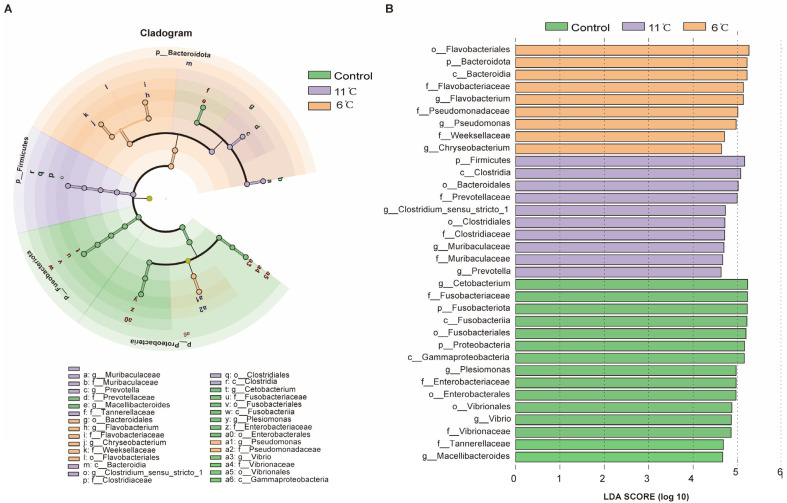
Taxonomic distribution and differential abundance of gut microbiota in *P. vachelli* under cold stress. (**A**) Cladogram showing the phylogenetic distribution of microbiota associated with three comparison groups. (**B**) Histogram of the LDA scores, where the LDA score indicates the effective size and ranking of each differentially abundant taxon. The taxa with Kruskal–Wallis < 0.05 and LDA > 4 are shown.

LefSe analysis was used to screen biomarkers with significant differences between groups from phylum level to genus level (LDA score > 4). At the phylum level, the cladogram revealed distinct microbial community structures among the experimental groups. Fusobacteriota was identified as the dominant phylum in the control group, while Firmicutes and Bacteroidota emerged as the predominant phyla in the low-temperature groups at 11 °C and 6 °C, respectively (Figure 7A). Linear Discriminant Analysis (LDA) identified 34 significantly enriched taxa in total, with differential distribution across groups: 15 taxa were enriched in the control group, 10 in the 11 °C low-temperature group, and 9 in the 6 °C low-temperature group (Figure 7B). At the genus level, the control group exhibited four unique microorganisms. *Muribaculaceae* and *Prevotella* were identified as biomarkers in the low-temperature (11 °C) group, which effectively distinguished this group from others. Meanwhile, the 6 °C low-temperature group was marked by the presence of three distinctive genera: *Flavobacterium*, *Pseudomonas*, and *Chryseobacterium*.

### 3.7. Functional Analysis of the Gut Microbiome in Pelteobagrus vachelli Under Low-Temperature Stress

**Figure 8:**
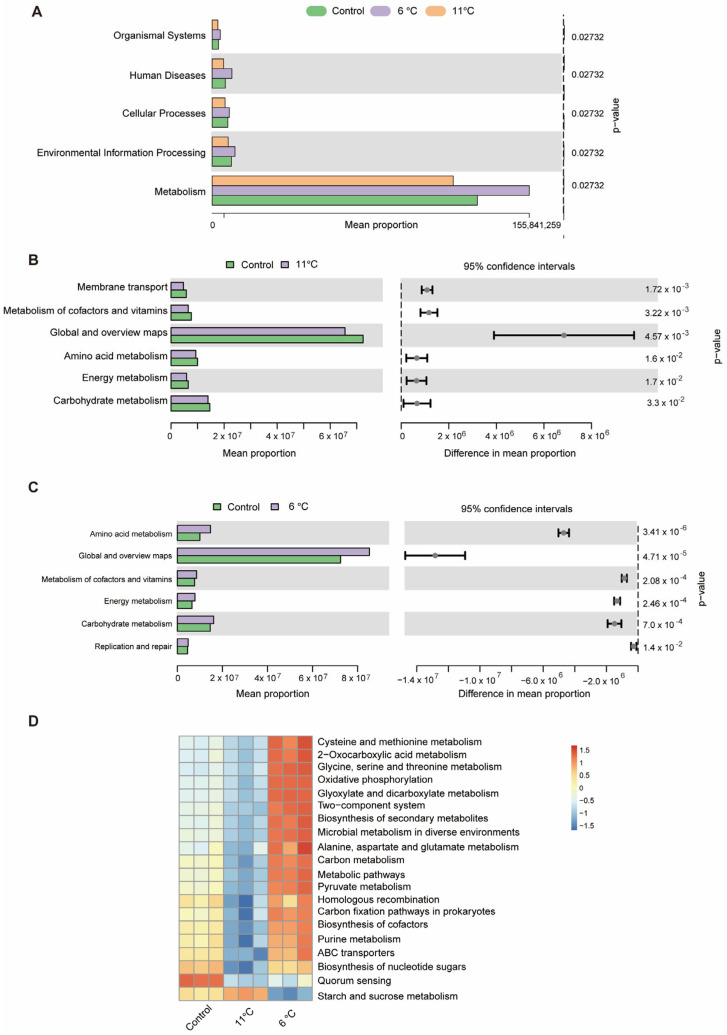
Microbiota function analysis of the KEGG pathways predicted by PICRUSt2. (**A**) Bar plot showing differential analysis pathways at the KEGG level 1 function. (**B**) *T*-test bar plot of significantly different pathways enriched at KEGG functional enrichment at level 2. (**C**) The abundance ratio of gut microbiota among the cold stress groups and control group predicted the level 2 function. (**D**) Relative abundance clustering heatmap at the KEGG level 3 function.

KEGG pathways showed that intestinal microbial functions of *P. vachelli* was altered by low-temperature stress (Figure 8). At the KEGG level 1, KEGG pathways enriched in all groups are classified into five categories: “Organismal system”, “Human diseases”, “Cellular processing”, “Environmental information processing”, and “Metabolism” (Figure 8A). Furthermore, at the KEGG level 2, compared with the control group, the pathways of “Amino acid metabolism”, “Carbohydrate metabolism”, “Energy metabolism”, and “Metabolism of cofactors and vitamins” pathways showed significant differences in the low-temperature stress groups (Figure 8B,C). At KEGG level 3, the clustering heat map derived from the KEGG analysis distinctly revealed the top 20 metabolic pathways that exhibited significant differences within the gut microbiota of *P. vachelli* when subjected to low-temperature stress (Figure 8D). Compared with the control group, the functional abundances of amino acid metabolism, energy metabolism, substance synthesis, substance transport, pathways of the flora in the 6 °C low-temperature group, such as “Cysteine and methionine metabolism”, “Alanine, aspartate and glutamate metabolism”, “Glycine, serine and threonine metabolism”, “ABC transporters”, and “Purine metabolism”, were significantly elevated. In contrast, in the 11 °C low-temperature group, these functional abundances were decreased. However, the starch and sucrose metabolism pathway were significantly increased in the 11 °C low-temperature group.

### 3.8. Correlation Analysis Between Liver and Intestine in Pelteobagrus vachelli

To elucidate the intricate relationships between gut microbiota abundance and hepatic phenotypic parameters/metabolites in *P. vachelli* under low-temperature stress, we performed Spearman correlation analysis. Our results demonstrated that cold exposure induced significant hepatic damage and metabolomic alterations, concomitant with disruption of intestinal microbial community structure.

**Figure 9:**
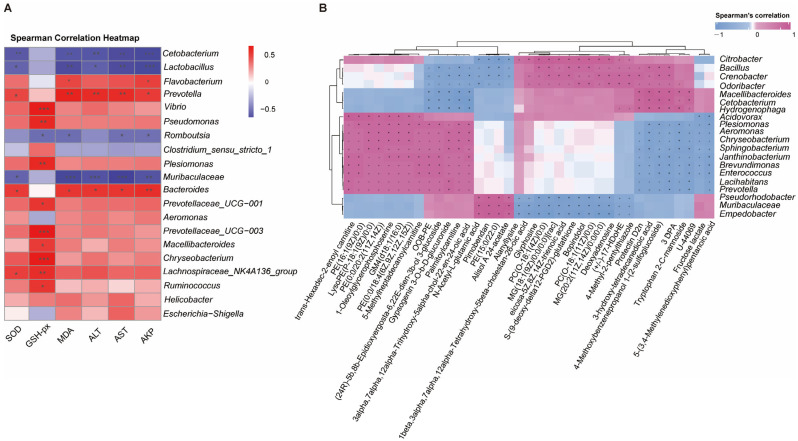
Correlation analysis between gut microbiota and liver tissue. (**A**) Sperman’s correlation analysis between the top 20 intestinal flora at the genus abundance and the physiological biomarkers (oxidative stress and immune indices) in the liver tissues. Asterisk indicates a significant difference, (* *p* < 0.05, ** *p* < 0.01, and *** *p* < 0.001), red represents positive correlations, while blue represents negative correlations. Color intensity is proportional to the extent of change. (**B**) Clustering heat map of top 30 differentially expressed metabolites (DEMs) in liver tissues and top 20 intestinal flora at genus abundance. Asterisk or plus indicates a significant difference. * *p* < 0.05, + *p* < 0.01), pink represents positive correlations, while blue represents negative correlations. Color intensity is proportional to the extent of change.

Notably, differential abundance analysis at the genus level revealed distinct correlation patterns with hepatic injury markers. The results showed that beneficial bacterial genera such as *Cetobacterium*, *Lactobacillus*, and *Muribaculaceae* exhibited significant negative correlations with the indices related to the antioxidant system (SOD, GSH-PX, MDA) and the liver injury indices (ALT, AST, AKP). Conversely, putative pathogenic genera, including *Flavobacterium*, *Prevotella*, and *Bacteroides*, exhibited statistically significant positive correlations with these hepatic parameters. Additionally, *Escherichia-Shigella*, *Helicobacter*, and *Lachnospiraceae_NK4A136_group* demonstrated a marginal positive association trend (Figure 9A).

Further analysis revealed significant correlations between specific bacterial genera and hepatic metabolites (Figure 9B). Notably, *Plesiomonas*, *Aeromonas*, *Enterococcus*, and *Prevotella* exhibited strong positive correlations with several acylcarnitines (e.g., trans-hexadec-2-enoyl carnitine, palmitoylcarnitine), phospholipid metabolites (e.g., LysoPE (P-18:1(9Z)/0:0), PE(0:0/20:2(11Z,14Z)), PC(O-18:1(11Z)/0:0)), and N-acetyl-L-glutamic acid. In contrast, these genera were negatively correlated with polyunsaturated fatty acid derivatives (e.g., (±)-17-HDoHE, eicosa-5Z,8Z,14Z-trienoic acid) and anti-inflammatory mediators (e.g., protectin D2n). Conversely, *Odoribacter*, *Bacillus*, and *Cetobacterium* displayed inverse association patterns, suggesting potential antagonistic interactions in host metabolic regulation. The findings suggest that gut microbiota–liver metabolic crosstalk may regulate *P. vachelli*’s response to cold stress.

## 4. Discussion

### 4.1. Dysregulation of Hepatic Antioxidant–Immune Homeostasis Under Low-Temperature Stress

Oxidative stress triggered by environmental stimuli serves as a critical physiological mechanism for organisms to cope with external stressors. Environmental challenges, like extreme temperatures, can severely disrupt redox homeostasis in aquatic organisms, leading to excessive generation of reactive oxygen species (ROS). This overproduction subsequently induces a cascade of oxidative damage effects, including protein oxidation, lipid peroxidation, and DNA lesions [34,35]. To counteract this threat, organisms have evolved sophisticated defense systems comprising both enzymatic antioxidants such as SOD, CAT, and GSH-Px and non-enzymatic antioxidants (e.g., glutathione). These coordinated systems maintain oxidative balance through efficient ROS scavenging mechanisms [36].

In this study, it was found that after exposure to low-temperature stress at 6 °C and 11 °C for 7 days, the activities of SOD and GSH-Px in the liver of *P. vachelli* increased significantly (Figure 3A,B). This finding is consistent with the results of previous studies on the Chinese softshell turtle (*Pelodiscus sinensis*) [37] and black porgy (*Acanthopagrus schlegelii*) [4], in which the activities of SOD and GSH-Px increased after low-temperature exposure. This result indicates that short-term low-temperature stress can maintain oxidative balance by upregulating the activities of antioxidant enzymes.

Notably, in the 6 °C group, the study observed decreased activities of SOD and GSH-Px compared to the 11 °C group, accompanied by continued accumulation of MDA (Figure 3C), a lipid peroxidation byproduct. This pattern aligns with the documented trends in zebrafish (*Danio rerio*) and whiteleg shrimp (*Litopenaeus vannamei*), which similarly exhibited reduced SOD activity and increased MDA levels following prolonged cold exposure [38,39]. Collectively, these data indicate that sustained stress beyond compensatory thresholds disrupts antioxidant synthesis/repair mechanisms, impairing ROS neutralization and initiating lipid peroxidation cascades, thereby amplifying cytotoxic MDA accumulation. Histopathological analyses revealed irreversible hepatic damage in the 6 °C group, characterized by disorganized hepatic cord architecture, cellular vacuolation, and nuclear pyknosis, indicating that redox homeostasis disruption serves as a critical pathogenic mechanism underlying organ injury.

The pathological escalation of oxidative injury was further validated by hepatic biomarker alterations. ALT and AST levels were significantly elevated in the low-temperature group compared with the control group (Figure 3E,F), indicating hepatic tissue damage—a pattern consistent with black porgy (*Acanthopagrus schlegelii*) exposed to cold stress [4]. Concurrently, cold stress induced a temporal surge in AKP activity (Figure 3D), mirroring responses documented in whiteleg shrimp (*Litopenaeus vannamei*) [40]. This suggests AKP may participate in damage repair through phosphate metabolism regulation, with its upregulated activity signifying both immune defense activation and metabolic homeostasis disruption.

### 4.2. Adaptive Reprogramming of Hepatic Metabolome and Metabolic Pathways Under Low-Temperature Stress

Our findings demonstrated that cold stress (6 °C and 11 °C) induced significant reorganization of hepatic metabolic signatures in *P. vachelli*, characterized by comprehensive reprogramming of amino acid metabolism, lipid metabolism, bile acid biosynthesis, and antioxidant-related pathways (Figure 5A,B). These metabolic network adaptations suggest a coordinated physiological strategy to mitigate cold-induced oxidative stress and energy deficit through metabolic homeostasis reconfiguration.

The marked downregulation of L-glutamate, a critical precursor for glutathione (GSH) biosynthesis, warrants particular attention in oxidative stress regulation. This reduction might impair hepatic GSH synthesis through suppression of γ-glutamylcysteine ligase (GCL) activity [41], a mechanism consistent with glutamate metabolic disturbance exacerbating oxidative damage in mammalian non-alcoholic fatty liver disease (NAFLD) models [42,43]. Concomitantly, the accumulation of lipid peroxidation end product 2E,13Z–Octadecadienal may directly activate the NF-κB-mediated inflammatory pathway and induce hepatocyte apoptosis [44], collectively amplifying hepatic oxidative injury. These findings mutually corroborate with the significant elevation of oxidative stress biomarkers observed in whiteleg shrimp (*Litopenaeus vannamei*) under low-temperature stress [45,46], strongly suggesting that redox imbalance constitutes a central challenge in aquatic ectotherms’ thermal adaptation mechanisms.

At the lipid metabolism level, a striking contrast exists between taurodeoxycholic acid (TUDCA) downregulation and phosphatidylcholine (PC) enrichment (Figure 5C,D). Reduced levels of TUDCA, a key hydrophilic bile acid, may disrupt cholesterol metabolism and promote intrahepatic cholestasis by impairing the Farnesoid X receptor (FXR) signaling pathway, thereby exacerbating hepatotoxicity [47]. Conversely, PC upregulation may mitigate cold-induced membrane phase transition damage by enhancing lipid bilayer fluidity and stability [48,49]. This bile acid metabolism sacrifice–membrane protection reinforcement strategy aligns closely with phospholipid remodeling during cold adaptation [50,51]. Notably, shared pathways such as ABC transporters and glycerophospholipid metabolism exhibited context-dependent roles in this process. At 11 °C, their upregulation facilitated detoxification and membrane remodeling for cold adaptation, whereas at 6 °C, energy limitation likely impaired active transport (reflected in ABC transporter downregulation) and induced purine catabolism for emergency energy supply. This divergence reflects a critical trade-off between adaptive plasticity (11 °C) and pathological survival (6 °C), underscoring the metabolic constraints imposed by severe cold stress.

KEGG enrichment analysis revealed that under low-temperature stress, the hepatic metabolic network of *P. vachelli* employs coordinated regulation through multiple pathways to counteract oxidative damage and energy imbalance. As a key modulator of temperature fluctuation adaptation in teleosts [52,53], FoxO signaling was significantly upregulated in the liver of *P. vachelli* under chronic cold stress, suggesting its conserved role in orchestrating compensatory responses to thermal challenges. This strategy aligns with observations in zebrafish (*Danio rerio*) [54] and carp family (*Cyprinidae*) [55]. Significant alterations in amino acid metabolism reflect energy substrate redistribution. Enhanced glutamate metabolism, for instance, may alleviate oxidative damage by generating precursors of glutathione, such as γ-glutamylcysteine [56], a mechanism critical for cold adaptation in white croaker (*Nibea albiflora*) [57]. Additionally, the upregulation of ABC transporters could alleviate hepatic burden by accelerating the efflux of bile acids and amino acid, consistent with findings on cold-induced detoxification in Oncorhynchus mykiss [49]. However, as observed in the context-dependent response, severe cold (6 °C) may override this protective mechanism, forcing a shift toward emergency energy mobilization via purine catabolism. Studies have demonstrated that purine metabolism critically mediates cold stress adaptation, as evidenced in white croaker (*Nibea albiflora*) [58], whiteleg shrimp (*Litopenaeus vannamei*) [57], and yellow catfish (*Pelteobagrus fulvidraco*) [21]. This evolutionary conservation implies *P. vachelli* likely employs analogous purine metabolic regulation to combat low-temperature challenges.

### 4.3. Low-Temperature Stress Induces Structural and Functional Disruption of the Gut Microbiota

The gut microbiota, recognized as the “second genome” in fish physiology, plays a pivotal role in maintaining host homeostasis through its involvement in nutrient metabolism, immunomodulation, and mucosal barrier protection [59]. Our results demonstrate a temperature-driven microbial shift characterized by decreased *Fusobacteriota* dominance and concurrent enrichment of *Firmicutes* and *Bacteroidota* under cold stress (Figure 7A). *Bacteroidetes* have been reported to be involved in carbohydrate transport and protein metabolism, which are important for the digestion of the diet [60], whereas *Firmicutes* are recognized as primary butyrate producers. Members of Firmicutes generate short-chain fatty acids (SCFAs) [61], particularly butyrate, which sustains intestinal mucosal homeostasis by energizing epithelial cells and modulating microbial community dynamics [62]. Consequently, we hypothesize that this temperature-responsive microbial transition in *P. vachelli* may facilitate energy compensation through accelerated nutrient transport and subsequent short-chain fatty acid (SCFAs) production, particularly butyrate and propionate, known for their thermogenic properties. Notably, gut-beneficial bacteria *Cetobacterium* a predominant acetate producer in control groups exhibited marked depletion under low-temperature conditions. This microbial restructuring aligns with reports in cold-exposed hybrid sturgeon (*Acipenser baerii* ♀ × *A. schrenkii* ♂) showing reduced *Cetobacterium* abundance [9]. The metabolites of *Cetobacterium* are rich in vitamin B12, which could promote the liver and gut health of fish [63,64]. A reduction of abundance of this probiotic may serve as an indicator of dysbiosis within intestinal microbiota. The observed *Cetobacterium* reduction could disrupt acetate-mediated energy homeostasis, potentially compromising cold tolerance mechanisms (Figure 6B,C). Of particular concern was the opportunistic proliferation of potential pathogens including Pseudomonas and Flavobacterium. These Gram-negative bacteria may exacerbate intestinal barrier dysfunction through lipopolysaccharide (LPS)-induced tight junction protein degradation [65], potentially initiating gut–liver axis-mediated hepatotoxicity [66,67].

Functional analysis revealed that low-temperature stress significantly altered intestinal microbial metabolic pathways. Notably, the 6 °C cold-exposed group exhibited marked upregulation of amino acid metabolism (e.g., cysteine and methionine metabolism) and ABC transporter pathways (Figure 8D), suggesting that gut microbiota may enhance host adaptation by improving nitrogen utilization efficiency and transmembrane transport processes to optimize nutrient absorption and allocation under cold-induced metabolic demands. This finding aligns with observations in *P. fulvidraco* under cold stress, where intestinal microbiota activated nitrogen metabolism pathways, indicating that microbial metabolic reprogramming represents a conserved strategy in teleosts to counteract energy deficits [21]. Conversely, the 11 °C group demonstrated an activation of starch and sucrose metabolism pathways, potentially reflecting host prioritization of carbohydrate catabolism for energy production under suboptimal thermal conditions. This metabolic shift corresponds with reported upregulation of glucose metabolism pathways in *Acanthopagrus schlegelii* cold adaptation models [4], implying that intestinal microbiota may assist hosts in reallocating energy resources through dynamic metabolic remodeling.

### 4.4. Low-Temperature Stress Perturbs Gut–Liver Axis Regulation

Many studies have demonstrated that cold stress induces gut microbial dysbiosis and concurrent damage to intestinal and hepatic tissues, with bidirectional interactions mediated through the gut–liver axis, a critical pathway linking intestinal microbiota to extraintestinal organs that plays a pivotal role in maintaining metabolic homeostasis [68]. The Spearman correlation analysis revealed statistically significant associations between specific gut bacterial taxa and hepatic injury biomarkers/metabolites, suggesting potential microbiota-driven regulation of liver function via metabolite exchange or immune signaling pathways. Notably, probiotic genera *Cetobacterium* and *Lactobacillus* exhibited negative correlations with antioxidant enzymes (SOD and GSH-PX), while showing inverse relationships with lipid peroxidation marker MDA and hepatocellular injury indicators (ALT and AST) (Figure 9A). This pattern implies their potential to mitigate oxidative stress through either direct secretion of glutathione precursors or modulation of endogenous antioxidant pathways. These findings align with mechanistic evidence from Musculus studies where Lactobacillus species attenuated hepatic oxidative damage via Nrf2 signaling activation [69]. Conversely, the positive correlation between the conditional pathogenic genera *Flavobacterium* and *Prevotella* and liver injury markers (Figure 9A) may reflect that they exacerbate the liver inflammatory response by releasing pro-inflammatory factors (such as LPS) or inhibiting the intestinal barrier function [65,70]. A similar mechanism has been reported in the gut–liver axis injury model of common carp (*Cyprinus carpio*) [71,72].

Further metabolomic analysis revealed that the opportunistic pathogens *Plesiomonas* and *Aeromonas* exhibited significant positive correlations with acylcarnitines (e.g., palmitoylcarnitine) and phospholipid metabolites (e.g., LysoPE) (Figure 9B), suggesting that these bacterial genera may induce hepatic lipid accumulation and membrane structural damage by interfering with mitochondrial β-oxidation or phospholipid remodeling processes [73,74]. Accumulation of acylcarnitines has been confirmed to be closely associated with mitochondrial dysfunction in mammalian non-alcoholic fatty liver disease (NAFLD) [75,76]. Additionally, the beneficial genus *Cetobacterium* was positively correlated with polyunsaturated fatty acid derivatives (e.g., 17-HDoHE) and the anti-inflammatory mediator Protectin D2n (Figure 9B). In this study, cold stress-induced reduction of *Cetobacterium* may attenuate its regulatory capacity to regulate host antioxidant and lipid metabolism pathways through decreasing acetic acid production, leading to an imbalance in the hepatic oxidative–antioxidative system and lipid accumulation, which exacerbated cold-induced liver inflammation. This microbiota–metabolite–liver phenotype cascading regulatory pattern is consistent with findings in Crucian carp (*Carassius carassius*) [77]. Therefore, our findings demonstrate that low-temperature stress induces significant alterations in the gut microbiota–host metabolism interplay in *P. vachelli*. This stress causes microbial dysbiosis: opportunistic pathogens multiply while beneficial commensals decrease. Intestinal homeostasis disruption leads to metabolic disorders and pro-inflammatory signaling, impairing the gut–liver axis and worsening hepatic injury.

## 5. Conclusions

**Figure 10:**
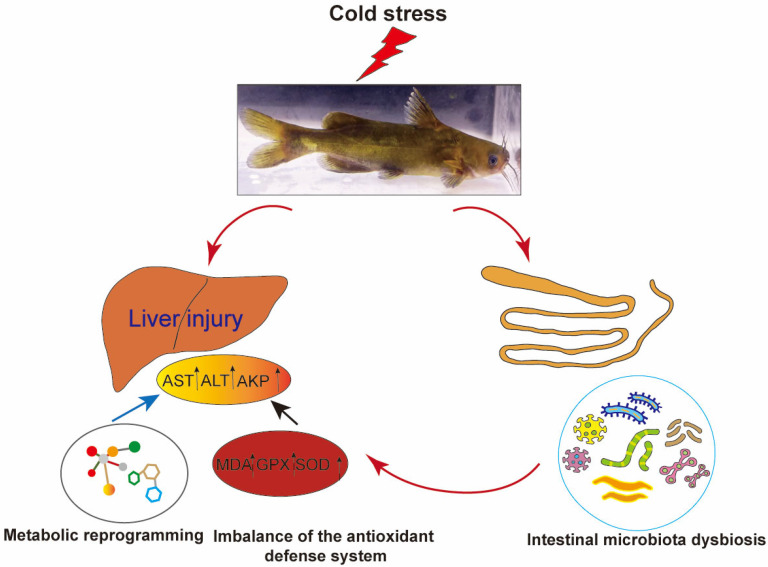
Low-temperature stress induced liver injury in *Pelteobagrus vachelli* via the gut–liver axis.

This study systematically investigated the cross-organ regulatory mechanisms of the gut–liver axis in *P. vachelli* under cold stress. The results demonstrated that acute cold exposure induced hepatic pathological damage, characterized by significantly elevated levels of AST and ALT, as well as an imbalance of the antioxidant defense system (evidenced by dynamic changes in SOD and GSH-Px activity, along with progressive MDA accumulation). Additionally, cold stress triggered the reprogramming of the FoxO signaling pathway, amino acid metabolism, glycerophospholipid metabolism, and purine metabolism. Therefore, low-temperature stress induces hepatic metabolic reprogramming in *P. vachelli* through multipathway interactions, manifested as dysregulation of the antioxidant defense system caused by metabolite imbalance, disruption of immune-related biosynthetic pathways, and imbalance in energy metabolism. Together, these effects disrupt hepatic metabolic homeostasis and induce tissue damage. Moreover, cold stress disrupted the composition of the intestinal microbial community of *P. vachelli*, marked by a significant increase in the abundance of pathogenic *Plesiomonas* and a corresponding decrease in beneficial *Cetobacterium*. Notably, these microbial shifts strongly correlated with hepatic injury biomarkers and metabolic perturbations, suggesting that intestinal dysbiosis may exacerbate cold-induced damage through metabolite-mediated gut–liver axis interaction (Figure 10).

This work provides the first comprehensive analysis of cold response mechanisms in *P. vachelli* from organ, metabolic, and microbial perspectives, offering a novel perspective for understanding the cold adaptation of fish. The findings establish a theoretical framework for developing innovative aquaculture strategies targeting microbiota modulation and metabolic interventions to enhance cold resistance.

## Data Availability

The data presented in this study are available on request from the corresponding author. The data are not publicly available due to being involved in another un-published project.

## Supplementary Materials

The following supporting information can be downloaded at: https://www.mdpi.com/article/10.3390/antiox14070762/s1, Figure S1: Experimental design flowchart; Figure S2: Metabolomic profiling: PLS-DA and differential metabolite distribution.; Figure S3: KEGG enrichment analyses of the DEGs in the cold stress and control comparison.
