# Supplementary material for: Low-Temperature Stress-Induced Hepatic Injury in Darkbarbel Catfish (Pelteobagrus vachelli): Mediated by Gut–Liver Axis Dysregulation
Source: Antioxidants (Basel). 2025 Jun 21;14(7):762. doi: 10.3390/antiox14070762 (PMC12291868; doi:10.3390/antiox14070762)
Supplement: Supplementary file 1 [file antioxidants-14-00762-s001.zip › antioxidants-3652978-supplementary.pdf]

Supplementary materials

Low-Temperature Stress-induced Hepatic Injury in Darkbarbel Catfish （*Pelteobagrus vachelli*）: Mediated by Gut-Liver Axis Dysregulation

Amei Liu[1,2], Guoqing Duan[1,2]*, Libo Yang[3], Yuting Hu[1,2], Huaxing Zhou[1,2], and Huan Wang[1,2]

1.Institute of Fisheries Science, Anhui Academy of Agricultural Sciences, Nongke South Road, Hefei, 230031, China; liuamei@aaas.org.cn (A. L.); duanguoqing@aaas.org.cn (G. D.); huyuting@aaas.org.cn (Y. H.); zhouhuaxing@aaas.org.cn (H. Z.); wanghuan@aaas.org.cn (H. W.)

2. Anhui Province Key Laboratory of Aquaculture & Stock Enhancement, Hefei, 230031, Anhui, China

3. Institute of Artificial Intelligence, Hefei Comprehensive National Science Center, Hefei 230088, Anhui, China; yanglibo1212@iai.ustc.edu.cn (L. Y.)

* Correspondence: duangq2010@126.com or duanguoqing@aaas.org.cn (D. G.); Tel: +86-0551-69116998

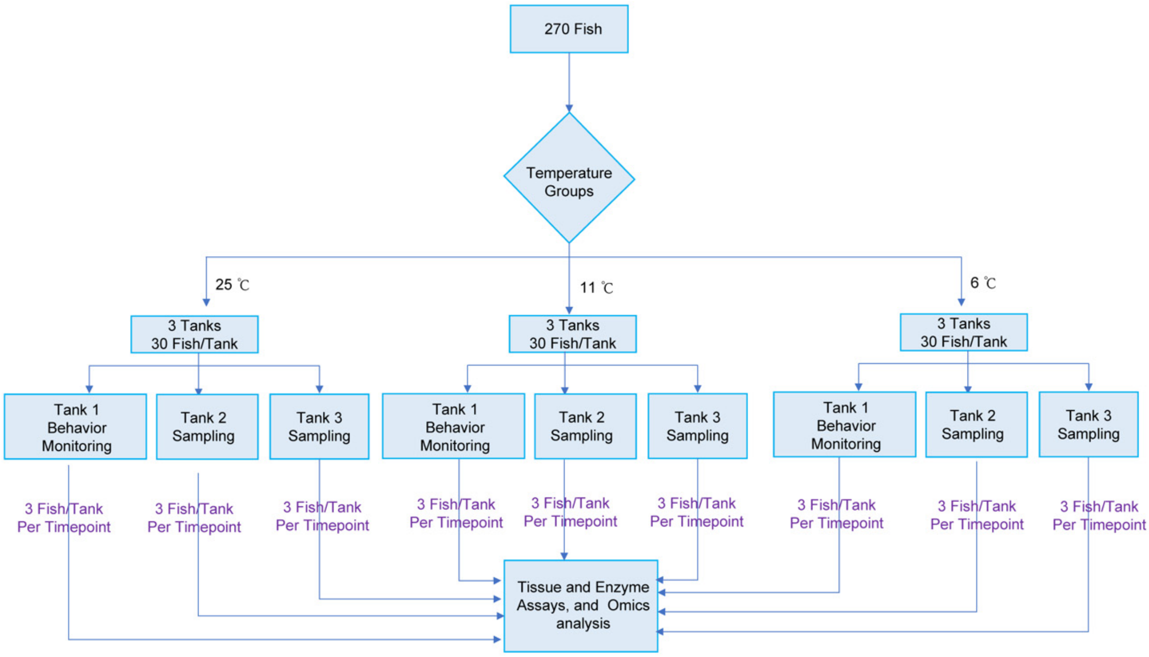

Figure supplement 1. Experimental design flowchart of cold stress treatment in *Pelteobagrus vachelli*.

A total of 270 *P.vachelli* were used in this study, which were randomly assigned to three

experimental groups with different temperature treatments: a control group maintained at 25℃ (ambient temperature), and two cold stress groups at 6℃and 11℃, respectively. Each group comprised three replicate aquariums (30 fish per tank), At the designated stress treatment time points, three fish were randomly sampled from each replicate aquarium within each group (n=3 per tank), leading to a total of nine fish sampled per group. Tissue collection was performed as follows: liver tissues were harvested for metabolomic analysis and biochemical assays, while intestinal segments were collected for microbial community analysis. Additionally, liver tissues from three randomly selected fish within each group were fixed in 4% paraformaldehyde for subsequent histological sectioning and hematoxylin-eosin (H&E) staining.

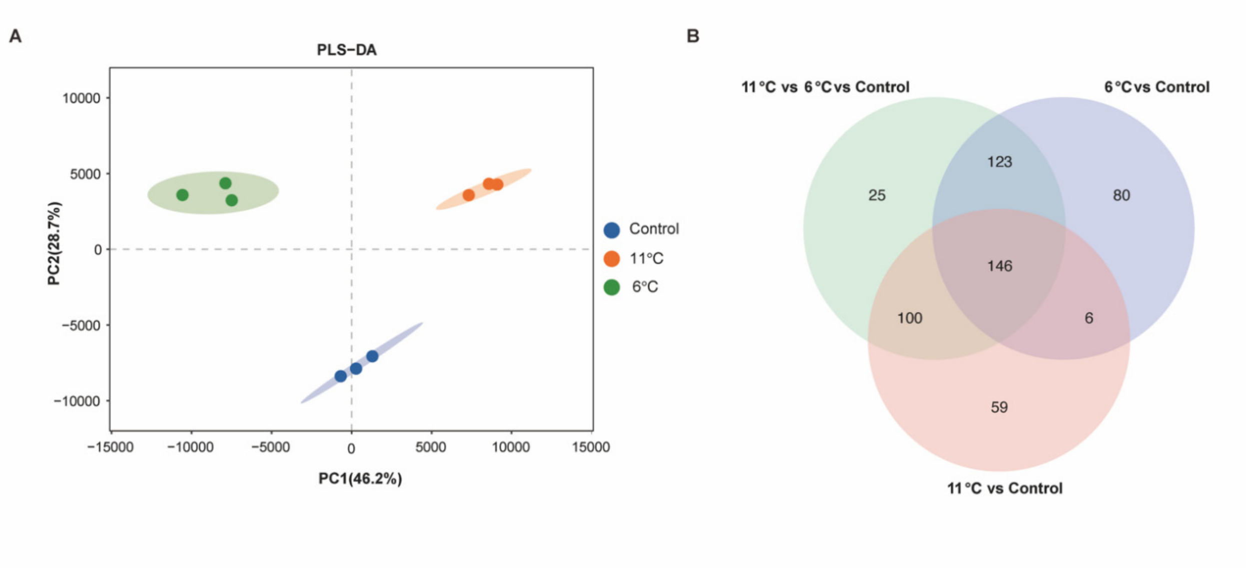

Figure supplement 2. Metabolomic profiling of *P.vachelli* under cold stress: PLS-DA and differential metabolite distribution.

(A) The partial least squares-discriminate analysis (PLS-DA). (B) Venn diagram of DEGs across three comparative groups.

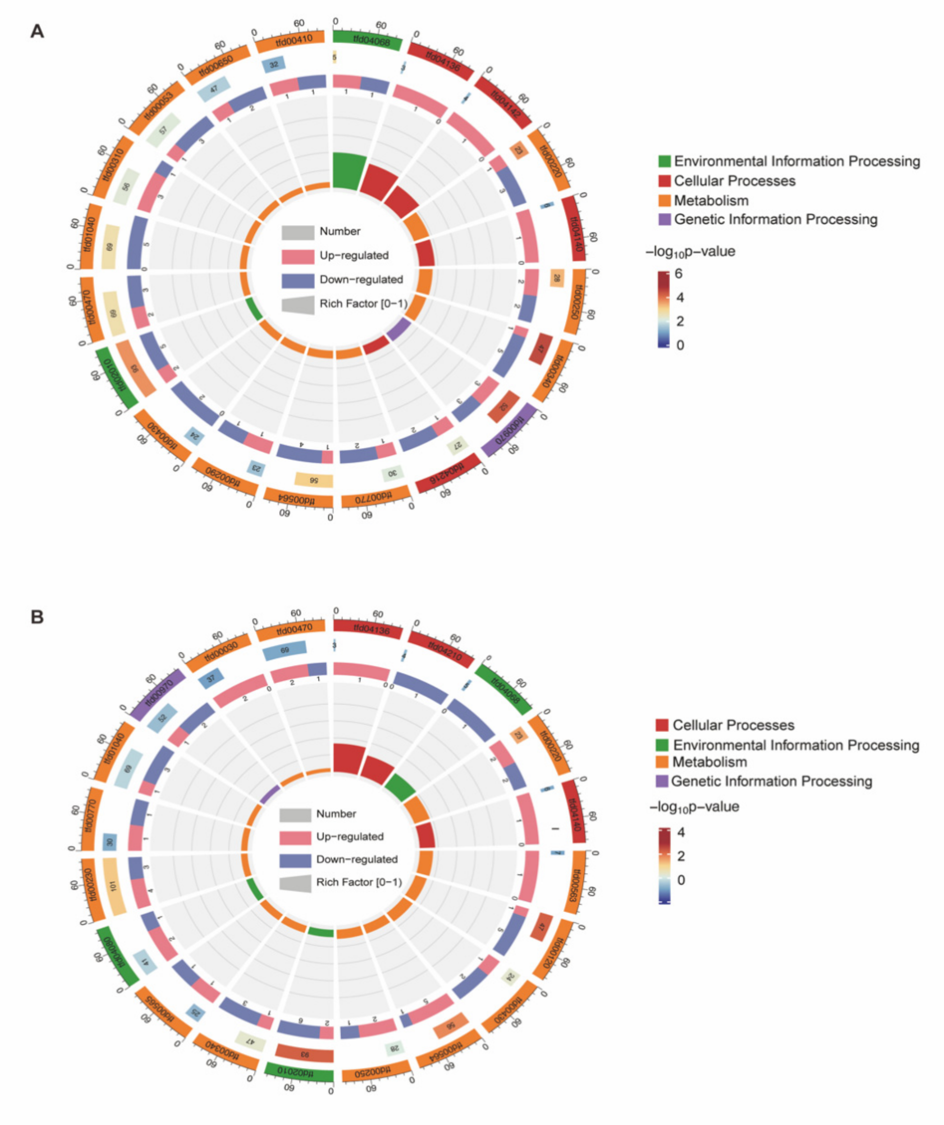

Figure supplement 3. KEGG enrichment analyses of the DEGs in the cold stress and control comparison. (A-B) Circos plot of KEGG enrichment analysis in three comparison groups DEGs. A, 11°C vs control, B, 6°C vs control.
